# Vesicular Stomatitis Virus Transcription Is Inhibited by TRIM69 in the Interferon-Induced Antiviral State

**DOI:** 10.1128/JVI.01372-19

**Published:** 2019-11-26

**Authors:** Tonya Kueck, Louis-Marie Bloyet, Elena Cassella, Trinity Zang, Fabian Schmidt, Vesna Brusic, Gergely Tekes, Owen Pornillos, Sean P. J. Whelan, Paul D. Bieniasz

**Affiliations:** a Laboratory of Retrovirology, The Rockefeller University, New York, New York, USA; b Department of Microbiology, Harvard Medical School, Boston, Massachusetts, USA; c Department of Molecular Physiology and Biological Physics, University of Virginia, Charlottesville, Virginia, USA; d Howard Hughes Medical Institute, The Rockefeller University, New York, New York, USA; University of Texas Southwestern Medical Center

**Keywords:** interferon, TRIM69, VSV

## Abstract

Interferons (IFNs) induce the expression of interferon-stimulated genes (ISGs), many of which are responsible for the cellular antiviral state in which the replication of numerous viruses is blocked. How the majority of individual ISGs inhibit the replication of particular viruses is unknown. We conducted a loss-of-function screen to identify genes required for the activity of alpha interferon (IFN-α) against vesicular stomatitis virus, Indiana serotype (VSV_IND_), a prototype negative-strand RNA virus. Our screen revealed that TRIM69, a member of the tripartite motif (TRIM) family of proteins, is a VSV_IND_ inhibitor. TRIM69 potently inhibited VSV_IND_ replication through a previously undescribed transcriptional inhibition mechanism. Specifically, TRIM69 physically associates with the VSV_IND_ phosphoprotein (P), requiring a specific peptide target sequence encoded therein. P is a cofactor for the viral polymerase and is required for viral RNA synthesis, as well as the assembly of replication compartments. By targeting P, TRIM69 inhibits pioneer transcription of the incoming virion-associated minus-strand RNA, thereby preventing the synthesis of viral mRNAs, and consequently impedes all downstream events in the VSV_IND_ replication cycle. Unlike some TRIM proteins, TRIM69 does not inhibit viral replication by inducing degradation of target viral proteins. Rather, higher-order TRIM69 multimerization is required for its antiviral activity, suggesting that TRIM69 functions by sequestration or anatomical disruption of the viral machinery required for VSV_IND_ RNA synthesis.

**IMPORTANCE** Interferons are important antiviral cytokines that work by inducing hundreds of host genes whose products inhibit the replication of many viruses. While the antiviral activity of interferon has long been known, the identities and mechanisms of action of most interferon-induced antiviral proteins remain to be discovered. We identified gene products that are important for the antiviral activity of interferon against vesicular stomatitis virus (VSV), a model virus that whose genome consists of a single RNA molecule with negative-sense polarity. We found that a particular antiviral protein, TRIM69, functions by a previously undescribed molecular mechanism. Specifically, TRIM69 interacts with and inhibits the function of a particular phosphoprotein (P) component of the viral transcription machinery, preventing the synthesis of viral messenger RNAs.

## Abstract (precis)

Interferons are important antiviral cytokines that work by inducing hundreds of host genes whose products inhibit the replication of many viruses. While the antiviral activity of interferon has long been known, the identities and mechanisms of action of most interferon-induced antiviral proteins remain to be discovered. We identified gene products that are important for the antiviral activity of interferon against vesicular stomatitis virus (VSV), a model virus that whose genome consists of a single RNA molecule with negative-sense polarity. We found that a particular antiviral protein, TRIM69, functions by a previously undescribed molecular mechanism. Specifically, TRIM69 interacts with and inhibits the function of a particular phosphoprotein (P) component of the viral transcription machinery, preventing the synthesis of viral messenger RNAs.

## INTRODUCTION

Infection of vertebrate animal cells by many viruses triggers innate immune responses, among which the induction of type I interferons (alpha/beta interferon [IFN-α/β]) is especially important. IFNs induce the expression of hundreds of interferon-stimulated genes (ISGs) that have a plethora of downstream effects in a wide range of cells (1). In particular, the products of many ISGs contribute to the establishment of the so-called “antiviral state,” in which the replication of many viruses is blocked (2, 3). Some ISGs have been shown to inhibit specific processes in viral replication or induce the destruction or depletion of viral proteins or RNA. However, the function of the majority of individual ISGs and precisely how they inhibit the replication of particular viruses remain unknown (4).

Vesicular stomatitis virus (VSV), a prototypic member of the order *Mononegavirales* and an important animal pathogen, is highly sensitive to inhibition by type I IFN. Like that of many RNA viruses, VSV replication occurs within specialized compartments in the cytoplasm of infected cells (5, 6). Compartmentalization may help to shield viral components from detection by cytosolic sensors or antiviral proteins that might otherwise increase IFN production or directly interfere with viral replication. For rhabdoviruses, such as VSV and rabies virus (RabV), and other cytoplasmically replicating negative-strand RNA viruses, replication compartments are not circumscribed by a membrane (6–9). Instead, replication components form inclusions that manifest features characteristic of phase-separated liquid compartments, such as P bodies and nucleoli (10, 11). Three VSV proteins, namely, the nucleoprotein (N), which coats the viral RNA, the large protein (L), which possesses all the viral enzymes necessary for transcription, and the phosphoprotein (P), which binds to both N and L to stimulate RNA synthesis, are necessary and sufficient for the assembly of phase-separated replication compartments (10). While not required for compartment formation, negative-strand viral RNA is located, replicated, and transcribed within these compartments once infection is established (6). However, the initial “pioneer” round of transcription, in which viral mRNAs are transcribed from a single incoming negative-strand viral genome, employs N, P, and L proteins that enter the cell as components of the incoming viral particle and, thus, occurs prior to the formation of phase-separated replication compartments.

Among the known antiviral proteins, a number of tripartite motif (TRIM) proteins have been shown to interfere directly with key steps in the life cycles of widely divergent viruses or exert indirect inhibition as regulators of antiviral signaling (12). While a variety of very distinct mechanisms and functions have been ascribed to TRIM proteins in this context, a characteristic feature of TRIM proteins is their shared architecture. Specifically, TRIM proteins form antiparallel dimers, driven by a central coiled-coil domain, that constitute one defining feature of the tripartite motif (13–15). At least some TRIM proteins also form higher-order multimers, mediated by interactions between N-terminal RING and/or B-box domains (16, 17), that are also defining features of the tripartite motif. Typically, SPRY or other protein domains situated at the TRIM protein C terminus enable interactions with viral or cellular targets (12). The propensity of TRIM proteins to form high-order multimeric structures thereby allows polyvalent interactions with targets.

Herein, we describe a loss-of-function screen to identify ISGs that are mediators of the anti-VSV activity of IFN-α. We show that the products of multiple ISGs, including previously unidentified antiviral proteins, contribute to the overall activity of IFN-α. Among these proteins, we identify a poorly characterized TRIM protein, TRIM69, as an inhibitor of VSV replication. We show that TRIM69 inhibits VSV replication through a previously unanticipated mechanism of action. Specifically, we find that higher-order TRIM69 multimers target a specific sequence in VSV P. In so doing, TRIM69 inhibits viral transcription and the formation of VSV replication compartments, resulting in profound reduction of viral RNA synthesis and inhibition of viral replication.

(This article was submitted to an online preprint archive [18].)

## RESULTS

### Identification of ISGs that mediate the anti-VSV activity of IFN-α.

**FIG 1:**
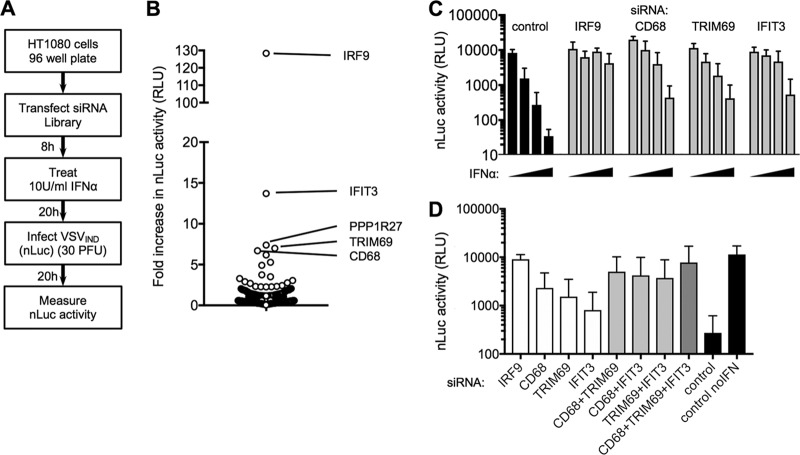
An siRNA-based screen for ISGs that inhibit VSV replication. (A) Schematic representation of the screening procedure for siRNA SMARTpools directed against ISGs. (B) VSV_IND_(nLuc) replication (fold increase in nLuc signal compared to the signal in the control) in cells transfected with siRNA SMARTpools (*n* = 400 SMARTpools). (C) Confirmatory assays of VSV_IND_(nLuc) replication (luciferase activity in relative light units [RLU]) in cells transfected with the indicated siRNA SMARTpools and treated with 0, 5, 10, or 20 U/ml of IFN-α. (D) VSV_IND_(nLuc) replication (luciferase activity in RLU) in HT1080 cells transfected with the indicated individual siRNAs, alone or in combination, and treated with 10 U/ml of IFN-α. Mean values ± standard deviations (SD) are shown.

To identify genes responsible for the antiviral state, specifically those responsible for inducing VSV (Indiana serotype [VSV_IND_]) resistance, we selected a subclone of HT1080 cells in which IFN potently inhibited the replication of a recombinant VSV_IND_ engineered to carry a nanoluciferase (nLuc) reporter gene [VSV_IND_(nLuc)]. We also designed a small interfering RNA (siRNA) library containing siRNA pools (Dharmacon SMARTpools containing 4 individual siRNA duplexes) representing the 400 most strongly upregulated genes among a panel of cell lines, along with 18 siRNA controls (Table 1). We transfected HT1080 cells in 96-well plates with the arrayed siRNA library and treated the cells with 10 U/ml IFN-α. The following day, cells were infected at a low multiplicity of infection (MOI) (0.01) with VSV_IND_(nLuc). After a further day, luciferase activity was measured, to identify siRNA pools that were able to enhance spreading VSV_IND_ replication in the presence of IFN-α (Fig. 1A).

**TABLE 1:** ISG siRNA SMARTpools included in siRNA screen

Product of siRNA SMARTpool target gene							
ABCD1	CD48	FAM195A	HS3ST3A1	LGALS1	NUB1	RGL1	STAP1
ACSL1	CD68	FAM198B	HSD11B1	LGALS3BP	NUP43	RGS1	STAT1
ACTA2	CD83	FAM46A	HSH2D	LGALS9	OAS1	RIN2	STAT2
ADAMDEC1	CD86	FAM89A	HSPA6	LHX2	OAS2	RNASEH2B	TAP1
ADAR	CDA	FAR2	IFI16	LILRB2	OAS3	RNF213	TAP2
ADRA1D	CEACAM1	FBXO30	IFI27	LILRB3	OASL	RSAD2	TARBP1
AGRN	CHCHD7	FBXO6	IFI30	LIN9	ODF2L	RTP4	TBX1
AHCTF1	CHMP5	FCER2	IFI35	LMO4	OSTM1	SAC3D1	TDRD7
AIM2	CHST12	FEZ2	IFI44	LRP8	OTOF	SAMD9	TGM1
AKAP2	CISD1	FRMD3	IFI44L	LY6E	OXR1	SAMD9L	TIMM9
ALYREF	CMPK2	FST	IFI6	LY96	PABPC1	SAP30	TM2D2
ANKFY1	CMYA5	GAL	IFIH1	LYAR	PARP10	SAR1B	TMED5
ANXA1	COA6	GBP1	IFIT1	LYSMD2	PARP12	SAT1	TMED7
ANXA3	COTL1	GBP2	IFIT2	MAD2L1	PARP14	SBNO2	TMEM123
APOL3	CPE	GBP3	IFIT3	MAFA	PARP9	SCOC	TMEM140
APOL6	CR2	GBP4	IFIT5	MAFB	PDGFRL	SDE2	TMEM245
ASCC3	CSAG1	GBP5	IFITM1	MAMLD1	PDHX	SDF4	TMEM259
ASF1A	CT45A4	GBP6	IFITM2	MAPK9	PGAP1	SDHAF3	TMEM55A
ATP5D	CT45A5	GCA	IFITM3	MASTL	PHF11	SDHD	TMEM62
AXL	CTSH	GCLM	IFNG	MBTPS2	PI4K2B	SEC24D	TNFSF10
B2M	CTSL	GFPT1	IGFBP3	MCM10	PIM1	SERINC1	TNFSF13B
BATF2	CUL1	GGH	IGSF1	METRNL	PITX1	SERPINE2	TNK2
BCL3	CXCL10	GIMAP2	IL1RAP	MMP9	PLEKHA4	SERPING1	TOR1B
BIN1	CXCL11	GIMAP4	IL1RN	MOV10	PLEKHO1	SFXN1	TRANK1
BLVRA	CXCL9	GIMAP7	IL27RA	MRPL55	PLGRKT	SGK1	TRIM21
BST2	CYBB	GMNN	IL4I1	MSRB3	PLSCR1	SGSH	TRIM22
BTG3	CYP1B1	GMPR	IL7R	MSX1	PML	SH2B2	TRIM25
C19orf66	CYP2J2	GNA13	IRF1	MT1A	PNKD	SHISA5	TRIM38
C1orf122	DAPP1	GNLY	IRF7	MTERF3	PNPT1	SIDT1	TRIM5
C1S	DDX58	GPATCH2	IRF8	MTFR1	PODXL2	SIGLEC1	TRIM69
C2orf47	DDX60	GPR65	IRF9	MTHFD1L	PPM1K	SLAMF8	TRMT13
C3orf58	DDX60L	GRINA	IRS1	MTSS1	PPP1R27	SLC15A3	TSNAX
C4orf33	DHX58	HAT1	ISG15	MVP	PPP2CB	SLC18B1	TTC21A
CA2	DLL1	HAVCR2	ISG20	MX1	PRDM2	SLC38A5	TTYH3
CARD17	DMRTA2	HELLS	JAK2	MX2	PRKAG2	SLC39A3	TYMP
CASP1	DNAJC24	HELZ2	JKAMP	MYD88	PRKD2	SLFN11	UBA7
CBFB	DOPEY1	HERC5	JUP	MYL4	PROCR	SLFN13	UBE2L6
CCDC146	DPYD	HERC6	KANK1	N4BP1	PRR5	SLFN5	UNC93B1
CCL13	DRAP1	HES4	KCTD12	NABP1	PSMB8	SNUPN	USP18
CCL2	DTX3L	HESX1	KIAA0020	NAPSA	PSMB9	SOAT1	USP41
CCL3L3	DYNLT1	HIGD1A	KIAA0101	NBN	PSME2	SOBP	VKORC1L1
CCL4L1	DYNLT3	HIST2H2AA3	KIAA0319L	NCOA7	PTMA	SOCS4	WARS
CCL4L2	EHD4	HLA-A	KLF4	NEXN	PTPN12	SP100	WDFY1
CCL8	EIF2AK2	HLA-B	KLF5	NFE2L2	RAB23	SP110	WSB1
CCR1	EMP1	HLA-C	KPNA3	NFE2L3	RAB8B	SP140	XAF1
CD14	ENDOD1	HLA-DRA	LAG3	NMI	RAI14	SPAG6	XRN1
CD163	ENPP2	HLA-E	LAMP3	NOP58	RARRES3	SPATS2L	YEATS4
CD164	EPSTI1	HLA-F	LAMTOR3	NPTX1	RASGRP3	SPI1	ZBP1
CD209	ETV7	HLA-G	LAP3	NRIP1	RBCK1	SPP1	ZFP36L1
CD38	FAM163A	HMOX1	LCLAT1	NT5C3A	REC8	SRM	ZNFX1

Using this approach, siRNA pools that increased VSV_IND_(nLuc) replication by >4-fold in both of two separate screens were identified (Fig. 1B). Upon retesting with fresh siRNA pools, IRF9, IFIT3, TRIM69, and CD68 were confirmed as *bona fide* hits, as the antiviral effect of IFN-α was clearly attenuated in siRNA-transfected cells (Fig. 1C). IRF9 was an expected hit, as it is required for type I IFN signaling, and transfection with siRNAs targeting IRF9 nearly completely abolished the inhibitory activity of IFN-α (Fig. 1C). Transfection of each siRNA individually or in combinations of two or three siRNAs suggested that IFIT3, CD69, and TRIM69 each contributed to the overall antiviral effect of IFN-α (Fig. 1D). Indeed, transfection of all three siRNAs in combination markedly diminished the activity of IFN-α (Fig. 1D).

### TRIM69 is an inhibitor of VSV_IND_ replication.

**FIG 2:**
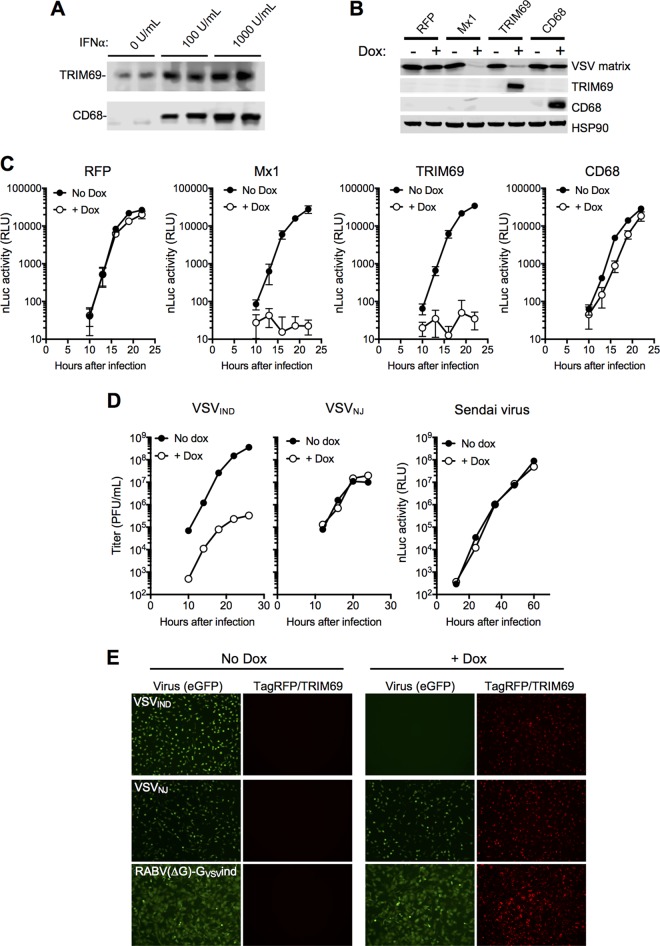
Overexpression of TRIM69 inhibits VSV_IND_ replication. (A) Western blot analysis of endogenous TRIM69 and CD68 expression following IFN-α treatment. (B) Western blot analysis of TRIM69, CD68, and VSV_IND_ M protein levels in HT1080 cells stably transduced with lentiviral vectors containing doxycycline (Dox)-inducible expression cassettes for the indicated genes, following induction with Dox and infection with VSV_IND_ for 5 h. (C) VSV_IND_(nLuc) replication (nanoluciferase [nluc] activity in RLU) in HT1080 cells stably transduced with lentiviral vectors containing doxycycline-inducible expression cassettes for the indicated genes and infected with 30 PFU VSV_IND_(nLuc) (MOI = 0.003). Mean values ± SD are shown. (D) VSV_IND_, VSV_NJ_, and Sendai virus replication in HT1080-TagRFP/TRIM69 cells, with or without doxycycline treatment. (E) HT1080-TagRFP/TRIM69 cells treated or not with doxycycline and infected with VSV_IND_(eGFP/P), VSV_NJ_(eGFP), or RabV(eGFP-ΔG)-G_VSVind_ 16 h later at an MOI of 1 for 1 h. Images were acquired at 6 h postinfection (h.p.i).

The IFIT family of proteins has previously been reported to recognize unusual structures at the ends of viral RNAs (19). We therefore focused our follow-up efforts on CD68 and TRIM69, which had not previously been reported to exhibit antiviral activity. Western blot analysis of HT1080 cells confirmed strong upregulation of both proteins upon IFN-α treatment (Fig. 2A). Using a doxycycline-responsive lentiviral expression vector, we established HT1080-derived cell lines that inducibly expressed CD68, TRIM69, or Mx1, a known anti-VSV protein, as a control (Fig. 2B). Doxycycline induction of TRIM69 or Mx1 expression potently inhibited VSV_IND_(nLuc) replication, while CD68 expression had only a modest inhibitory effect (Fig. 2C). Profound inhibition of replication was evident when VSV_IND_(nLuc) or unmodified VSV_IND_ was used at a low MOI to initiate spreading replication assays (Fig. 2C and D). In the latter case, there was an ∼1,000-fold reduction in the yield of infectious VSV_IND_ particles following a 26-h multicycle spreading replication experiment (Fig. 2D). When VSV_IND_ carrying the enhanced green fluorescent protein gene [VSV_IND_(eGFP)] was used in a short-term experiment (MOI = 1, 6 h [∼1 replication cycle]), the number of GFP-positive cells was clearly reduced (Fig. 2E). These data suggest that TRIM69 inhibits a step in the VSV_IND_ replication cycle prior to the assembly and release of infectious particles. Consistent with that conclusion, infection of HT1080 cells with VSV_IND_ and Western blot analysis 6 h later revealed that VSV_IND_ matrix (M) protein expression was profoundly inhibited by TRIM69 (Fig. 2B). We note that doxycycline-inducible TRIM69 was overexpressed compared to the expression of endogenous TRIM69 in these experiments, which likely increases the magnitude of its antiviral effect compared to that of endogenous TRIM69. Nevertheless, with this *caveat*, this single ISG was capable of profound inhibition of VSV_IND_ replication in the absence of the induction of other ISGs.

**FIG 3:**
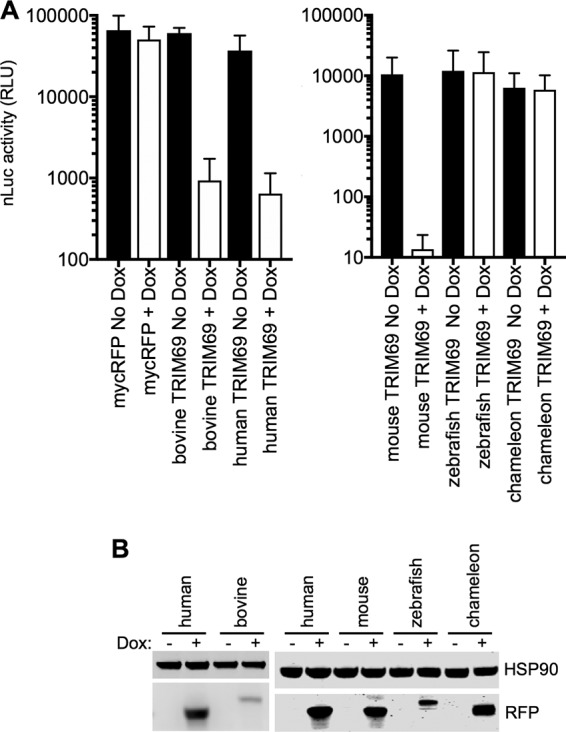
Antiviral activity of TRIM69 proteins from various species. (A) VSV_IND_(nLuc) replication in HT1080 cells expressing doxycycline-inducible myc-tagged TRIM69 proteins from various species. Mean values ± SD are shown. (B) Western blot analysis of TRIM69-myc protein expression following induction with doxycycline.

We tested whether the replication of other distantly or closely related negative-strand RNA viruses was inhibited by TRIM69. Neither Sendai virus (SeV), rabies virus, nor even the New Jersey strain of VSV (VSV_NJ_), which is ∼36% divergent from VSV_IND_, was inhibited by TRIM69 (Fig. 2D and E). Notably, TRIM69 proteins from two divergent mammalian (murine and bovine) species were active inhibitors of VSV_IND_, but TRIM69 proteins from more divergent species, a reptile (chameleon) and a fish (zebrafish), did not inhibit replication (Fig. 3A and B).

### Anti-VSV_IND_ activity is associated with high-order TRIM69 multimerization.

**FIG 4:**
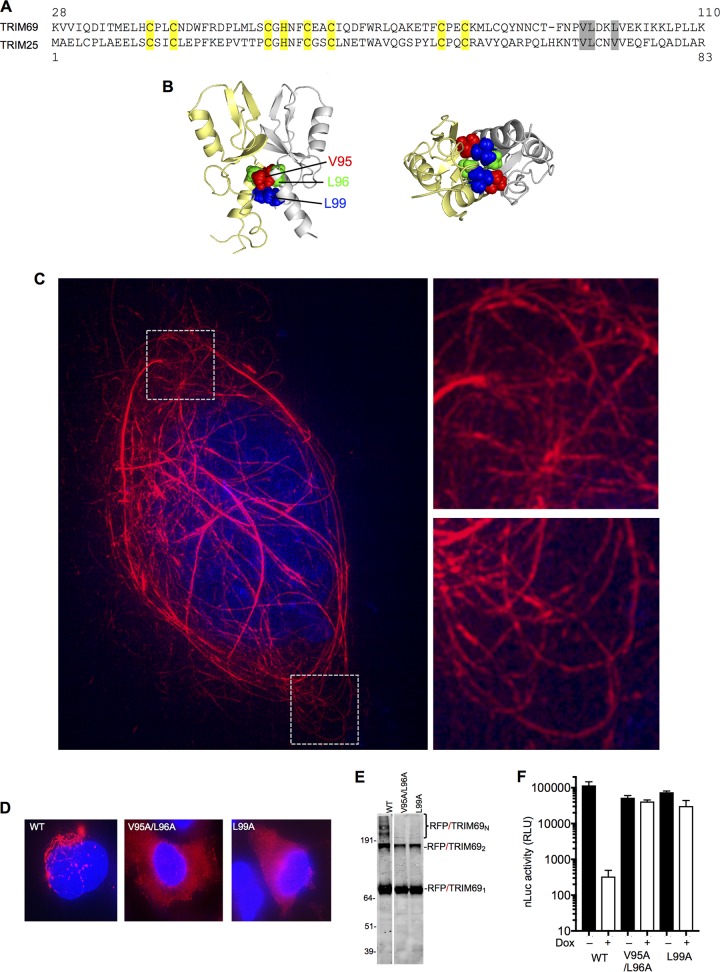
Requirement for TRIM69 multimerization for antiviral activity. (A) Alignment of TRIM69 and TRIM25 RING domains. Yellow, zinc-coordinating residues; gray, mutated residues at the dimer interface. (B) Crystal structure of dimeric TRIM25 RING domain indicating amino acids at the dimer interface: V68, L69, and V72 are analogous to V95, L96, and L99 in TRIM69. (C) 3-D–SIM image of an mScarlet-TRIM69 expressing cell, with expanded views of the boxed areas. (D) Deconvolution microscopic images of WT and mutant TagRFP/TRIM69 fusion proteins expressed in doxycycline-inducible HT1080 cells. (E) Western blot analysis of WT and mutant TagRFP/TRIM69 fusion proteins following treatment of cells with EGS cross-linker prior to cell lysis. (F) VSV_IND_(nLuc) replication (luciferase activity in RLU) in HT1080 cells stably transduced with lentiviral vectors containing doxycycline-inducible expression cassettes for WT and mutant TagRFP/TRIM69 proteins. Mean values ± SD are shown.

TRIM69 is a member of the large family of TRIM proteins, several of which have been shown to exhibit direct or indirect antiviral activity (12). Like other TRIM proteins, TRIM69 protein forms dimers that are held together by a coiled-coil domain, configured in antiparallel orientation (15). In addition, for at least some TRIM proteins, the anti-parallel dimers are assembled into higher-order multimeric structures, which are held together via a second dimer or trimer interface, located in the RING or B-box domains, respectively (16, 17). Among other TRIM proteins, TRIM25 contains a dimeric interface in its RING domain and also shares the highest level of sequence similarity with TRIM69 (Fig. 4A). We used the previously determined crystal structure of a TRIM25 RING domain dimer (17) to identify amino acids at the RING domain dimer interface (V95, L96, and L99 in TRIM69, analogous to V68, L69, and V72 in TRIM25) (Fig. 4A and B) whose mutation might disrupt the TRIM69 RING dimer interface and, perhaps, higher-order TRIM69 multimerization (Fig. 4B).

Three-dimensional structured illumination microscopy (3-D–SIM) superresolution imaging of mScarlet red fluorescent protein (RFP)-tagged TRIM69 (mScarlet/TRIM69) (Fig. 4C), as well as standard deconvolution microscopic imaging of TagRFP/TRIM69 expressed in HT1080 cells (Fig. 4D), showed that unmodified TRIM69 formed filamentous structures and cytoplasmic bodies in the cytoplasm of cells, consistent with the notion that it assembles into high-order structures. Moreover, Western blot analysis of cell lysates generated after treatment of cells with the protein cross-linker EGS [ethylene glycol bis(succininic acid *N*-hydroxysuccinimide ester)] showed that TRIM69 formed dimers and, apparently, higher-order multimers (Fig. 4E). Notably, mutation of the predicted RING domain dimer interface (a V95A L96A double mutant [bearing changes of V to A at position 95 and L to A at position 96] and an L99A single mutant) resulted in TagRFP/TRIM69 proteins that formed cross-linkable dimers but not higher-order multimers (Fig. 4E) and exhibited a diffuse rather than filamentous distribution in the cell cytoplasm (Fig. 4D). Notably, V95A L96A and L99A mutations abolished the ability of TRIM69 to inhibit VSV_IND_(nLuc) replication, suggesting that higher-order multimerization is necessary for antiviral activity (Fig. 4F).

### Recruitment of TRIM69 to sites of VSV replication and inhibition of replication compartment formation.

**FIG 5:**
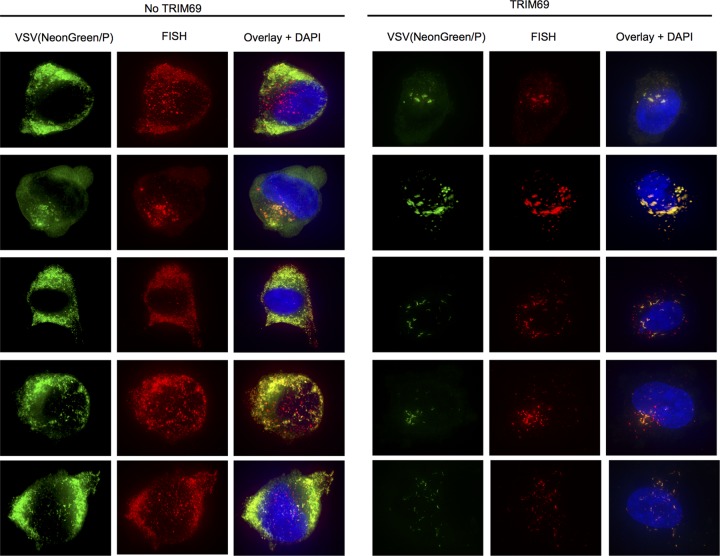
TRIM69 inhibits generation of VSV_IND_ replication compartments. Gallery of five randomly selected cells expressing (right) or not expressing (left) TRIM69, fixed 4 h after infection with VSV_IND_(NeonGreen/P) and subjected to FISH with probes targeting the negative-strand VSV RNA (N gene).

**FIG 6:**
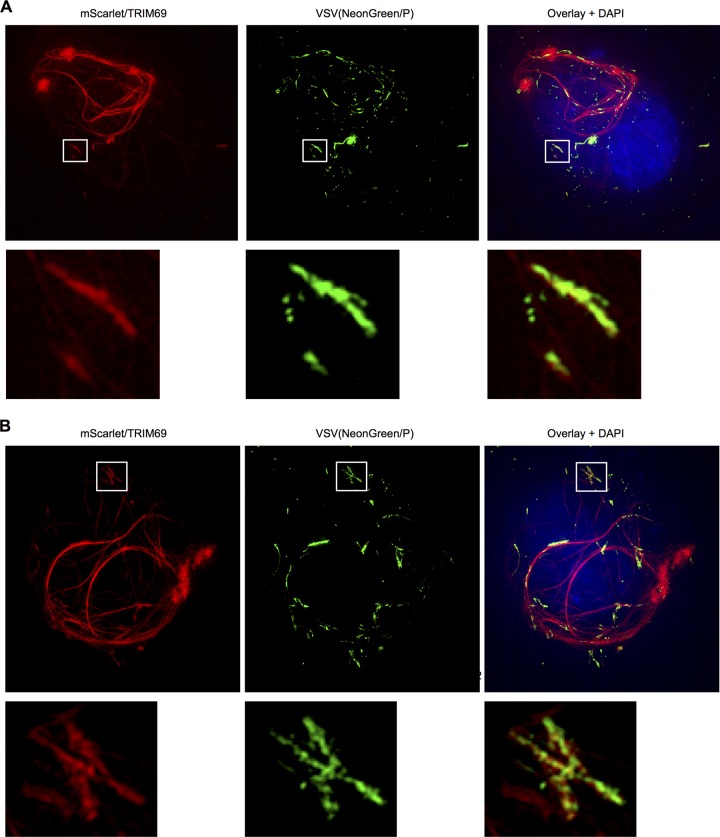
Association of TRIM69 with aberrant VSV_IND_ replication compartments. 3-D–SIM images of two mScarlet-TRIM69-expressing cells infected with VSV_IND_(NeonGreen/P), with expanded views of the boxed areas.

Like many RNA viruses, VSV partitions its replication machinery into specialized compartments within which RNA synthesis occurs (6–9). In the case of VSV, recent work has shown that these compartments are liquid inclusions that are not bounded by membranes but instead exhibit characteristics of phase separation (10). Replication compartments are conveniently labeled and localized using VSV clones modified to append a fluorescent protein to the amino terminus of P. We infected cells with VSV_IND_(NeonGreen/P) and visualized replication compartments using NeonGreen green fluorescent protein (GFP), along with fluorescence *in situ* hybridization (FISH) probes directed to the negative-strand viral RNA (Table 2). This analysis showed that the presence of TRIM69 profoundly attenuated the formation of replication compartments marked by the P protein and negative-strand RNA (Fig. 5). Additionally, 3-D–SIM imaging of cells expressing mScarlet/TRIM69 and infected with VSV_IND_(NeonGreen/P) revealed that the smaller P accumulations that were observed in TRIM69-expressing cells were typically colocalized with the mScarlet/TRIM69 filaments (Fig. 6). Indeed, many of the P accumulations appeared to adopt an elongated, almost filamentous structure, different in shape and size from the compartments typically observed in VSV-infected cells, and coincident with TRIM69 filaments (Fig. 6). Overall, these data suggested that a viral or cellular component governing the formation of viral replication compartments associates with TRIM69 and that this association ultimately inhibits replication compartment formation.

**TABLE 2:** FISH probes targeting VSV_IND_ N positive and negative RNA strands

Probe no.	N RNA strand targeted	
	Positive	Negative
1	TCT CTT GAC TGT AAC AGA CA	GGC AAG TAT GCT AAG TCA GA
2	GGA ACT ACG ACT GTG TTG TC	GCA AGG CCT AAG AGA GAA GA
3	ATC CTC ATT TGC AGG AAG TT	GCG AAA AGA GCA GTC ATG TC
4	AAG TAA TCT GCC GGG TAT TC	GGA TTG ACG ACT AAT GCA CC
5	GAG GAA TCT CCT TTG ATT TT	ACA CTC CAG ATG ATA GTA CC
6	GGT AGA CAT ATC CTC TTA GA	CGA CTT GGC ACA ACA GTT TT
7	TAC ATT TCC GGA TTT GAG GC	TTG TAC GCT TAT GCA GTA GG
8	GTA GCT GTT GAC ATG TAT GA	ATC TCT TAC TAC AGC AGG TT
9	ATG TCC TTT AAT GCT CCA TA	GAC AGC CTG ATG ACA TTG AG
10	CCC GAT GTT TAT TCC GAA AC	TCC ACC AGA GCA AGG AAT GC
11	AAT ATT CCG ATT GTA TCC CC	ATT GAC AGC TCT TCT GCT CA
12	GGG CTT TCA AGG ATA CAA GG	TTC TTC CGT CAA AAA CCC TG
13	CGA TAC TCC ATC TGG AAG TA	GAT TGT CTT CTA AGT CTC CA
14	AAG GCA ACC ATT TGT CAT CT	ATG CCT TAT TTG ATC GAC TT
15	CAC TCT GTA TAA GCC AAG TA	AAT TGA CAA GGC CGA TTC AT
16	ATT CAG GCA TTT GTG TTC TG	AAA TGA TGC TTC CAG GCC AA
17	CCA TCC ATG AGC TTT TTT CT	CCG AGA AGT TGC AGA TGA AA
18	TGT TCA TTG ATC ATT TTG CA	ATG TAA CGA CCT GGA TCT TG
19	TTC TGG CAC AAG AGG TTC AA	ATA ACC GGA ATG TCT ACA GA
20	TGC AGC GAC AAT TTT TGT GT	ATT GGC AAC ATT TGG ACA CC
21	AGT TCC GTA TCT GAA CGA GG	CCA GAT TCA AAG ATT GTG CT
22	GCA GCA CAA TCT TTG AAT CT	CCT CGT TCA GAT ACG GAA CT
23	GCA GAG GTG TCC AAA TGT TG	AAA ATT GTC GCT GCA GTG GA
24	CTG TAG ACA TTC CGG TTA TT	ACA GTT TGA ACC TCT TGT GC
25	AAG ATC CAG GTC GTT ACA TC	CTC ATG GAT GGG CTG ACA AA
26	TTT CAT CTG CAA CTT CTC GG	TAC AGA GTG GGC AGA ACA CA
27	CCT GGA AGC ATC ATT TGG AC	GTT GCC TTT GTA TCT ACT TG
28	GAA TCG GCC TTG TCA ATT TC	AGA ACC AGC GCA GAT GAC AA
29	GTC GAT CAA ATA AGG CAT GT	AGA TGG AGT ATC GGA TGC TT
30	GGA GAC TTA GAA GAC AAT CC	TAT CCT TGA AAG CCC TGG AC
31	GCA GGG TTT TTG ACG GAA GA	ACA ATC GGA ATA TTT GAC CT
32	GGT GGA TCT GAG CAG AAG AG	AAA CAT CGG GAA AGC AGG GG
33	ACT CAA TGT CAT CAG GCT GT	AAG ATT GGT CAA GTT TCG GA
34	CCT GCT GTA GTA AGA GAT GT	ATT AAA GGA CAT CCG GGG TA
35	CCT ACT GCA TAA GCG TAC AA	GTC AAC AGC TAC TTG TAT GG
36	AAA CTG TTG TGC CAA GTC GG	GCC TCA AAT CCG GAA ATG TA
37	CGG TAC TAT CAT CTG GAG TG	CTA AGA GGA TAT GTC TAC CA
38	CGG TGC ATT AGT CGT CAA TC	GAG ATT CCT CTT TAC ATC AA
39	GTG ACA TGA CTG CTC TTT TC	AGT GGA ATA CCC GGC AGA TT
40	ATT GTC TTC TCT CTT AGG CC	AAC TTC CTG CAA ATG AGG AT
41	TTC TGA CTT AGC ATA CTT GC	GAC AAC ACA GTC GTA GTT CC
42		TGT CTG TTA CAG TCA AGA GA

### The VSV phosphoprotein (P) is the viral determinant of TRIM69 sensitivity.

**FIG 7:**
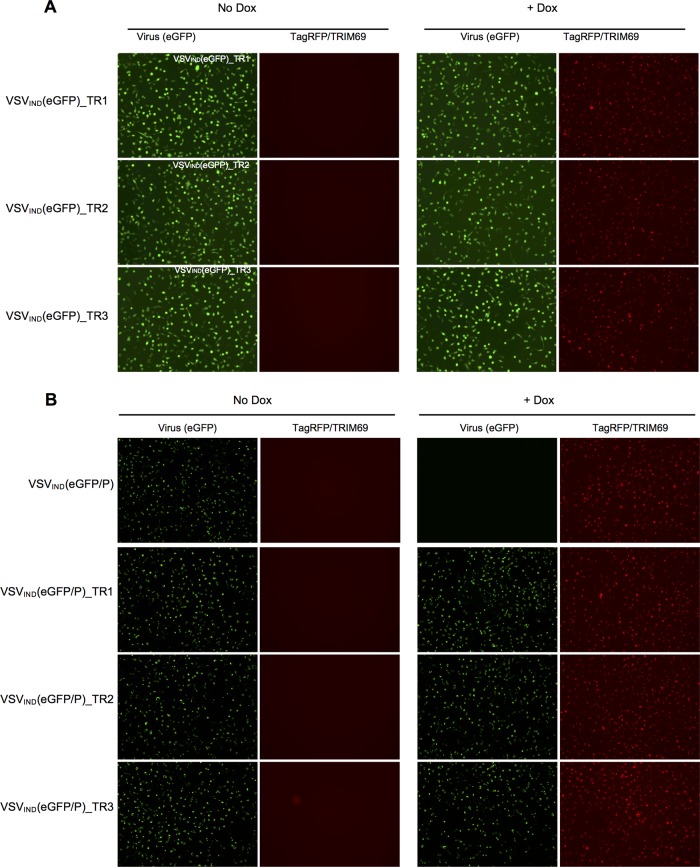
Derivation and characterization of TRIM69-resistant (TR) viruses. (A, B) HT1080-TagRFP/TRIM69 cells were seeded and simultaneously treated or not with doxycycline. Sixteen hours later, cells were infected with VSV_IND_(eGFP) (A) or VSV_IND_(eGFP/P) (B) TR clones at an MOI of 1 for 1 h. Images were acquired at 6 h.p.i.

**FIG 8:**
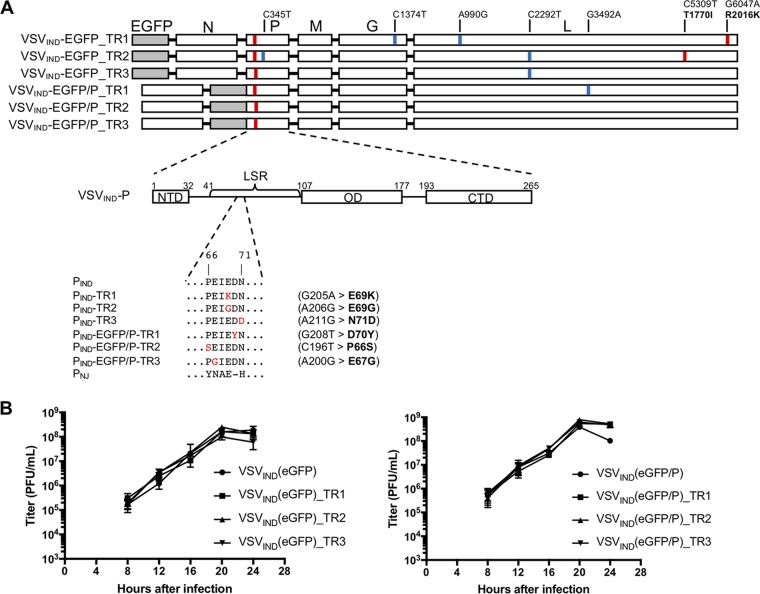
The VSV-P protein determines sensitivity to TRIM69. (A) Complete genome sequences of TRIM69-resistant viruses. Synonymous substitutions are indicated in blue, nonsynonymous mutations are indicated in red. The schematic of VSV_IND_ P shows its modular organization into three domains: the N-terminal domain (NTD), oligomerization domain (OD), and C-terminal domain (CTD). The L-stimulatory region (LSR, amino acids [aa] 41 to 106) and the peptide containing the point mutations found in TRIM69-resistant clones (aa 66 to 71) are indicated. All TR viruses contained a nonsynonymous mutation in the P (aa 66 to 71) peptide. (B) Replication of WT and TR VSV_IND_(eGFP) (left) or VSV_IND_(eGFP/P) (right) viruses in Vero cells infected at an MOI of 0.05. Mean values ± SD are shown.

To determine how TRIM69 inhibits VSV replication, we selected mutant VSV derivatives that were resistant to TagRFP/TRIM69. We infected HT1080 cells expressing TagRFP/TRIM69 with VSV_IND_(eGFP) or a VSV_IND_ clone in which eGFP was appended to P [VSV_IND_(eGFP/P)] at a high multiplicity (MOI = 10). Three growing plaques each were picked for VSV_IND_(eGFP) and VSV_IND_(eGFP/P) and amplified on HT1080 cells expressing TagRFP/TRIM69. These viruses infected HT1080 cells with equivalent efficiency whether TagRFP/TRIM69 was induced or not (Fig. 7A and B). Sequencing of the viral genome of these TRIM69-resistant (TR) viruses showed that all six encoded a nonsynonymous mutation within a short peptide sequence (amino acids 66 to 71) within P [P(66–71)] (Fig. 8A). For 2/6 viruses, the only mutations present were nonsynonymous changes in the P(66–71) peptide; in another 2/6 viruses, additional synonymous mutations were present; while in a further 2/6 viruses, additional nonsynonymous mutations were found. Based on these findings, we concluded that the P(66–71) mutations were likely responsible for the resistance to TRIM69 (Fig. 8A). This peptide sequence is within a region of P that contacts the globular connector, methyl transferase, and C-terminal domain (CTD) of L and is also responsible for stimulating polymerase activity (the L-stimulatory region [LSR]) (Fig. 8A) (20, 21). Although located in a region crucial for efficient viral RNA synthesis, the TR mutations did not affect replication of VSV_IND_(eGFP) or VSV_IND_(eGFP/P) in Vero cells (Fig. 8B). Notably, the corresponding amino-acid-66 to -71 sequence in the P protein of VSV_NJ_ is different in 5/6 amino acid positions (Fig. 8A), providing a potential explanation for the intrinsic resistance of VSV_NJ_ to TRIM69.

### TRIM69 associates with VSV_IND_ P.

**FIG 9:**
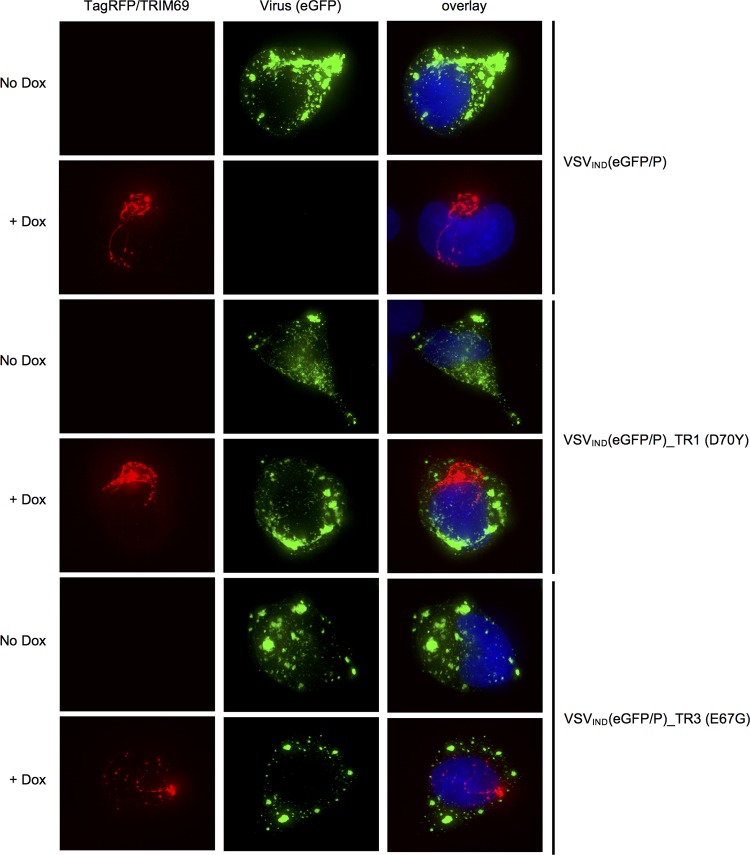
Replication compartments form independently of TRIM69 in TR virus-infected cells. HT1080-TagRFP/TRIM69 cells were treated or not treated with doxycycline and infected with VSV_IND_(eGFP/P) and TRIM69-resistant mutants (D70Y or E67G) thereof. Images were acquired at 4 h.p.i.

**FIG 10:**
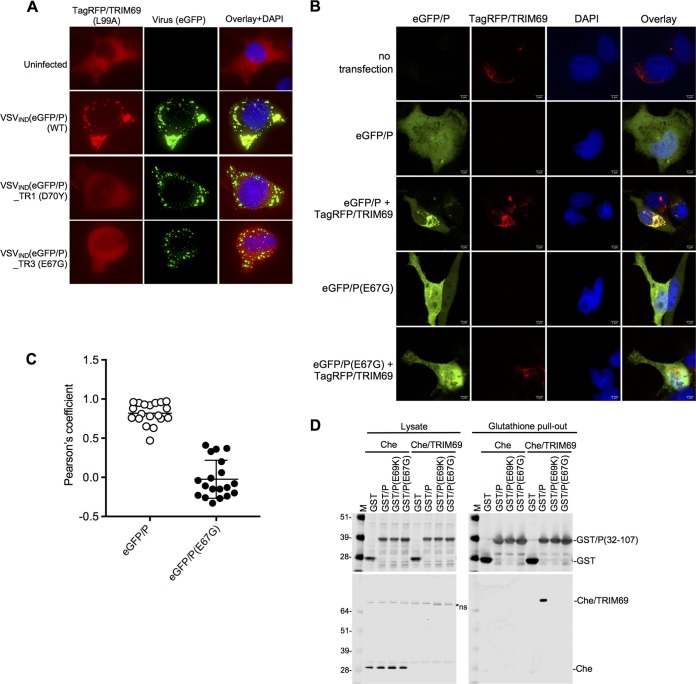
TRIM69 binds to P. (A) Deconvolution microscopic images of HT1080-TagRFP(L99A) mutant cells infected with WT or mutant VSV_IND_(eGFP/P). (B) HT1080-TagRFP/TRIM69 cells were seeded and simultaneously treated or not with doxycycline. Sixteen hours later, cells were transfected or not with plasmids expressing eGFP/P or eGFP/P(E67G). Twenty-four hours posttransfection, cells were fixed, stained with DAPI, and imaged with a spinning-disk confocal microscope. (C) Colocalization analysis of eGFP/P and TagRFP/TRIM69 using ImageJ software. Unpaired *t* test, *n* = 19, *P* < 0.0001. Mean values ± SD are shown. (D) Western blot analyses (top, anti-GST antibody; bottom, anti-Cherry fluorescent protein antibody) of cell lysates and glutathione pull-out fractions from 293T cells transfected with plasmids expressing Cherry/TRIM69 and either WT or mutant GST-P proteins.

We next investigated how the TR mutations in P exerted their effects. While wild-type (WT) TagRFP/TRIM69 effectively prevented the formation of VSV_IND_(eGFP/P) replication compartments, the two VSV_IND_(eGFP/P) TR mutants, SVIND(eGFP/P)_TR1(D70Y) and VSVIND(eGFP/P)_TR3(E67G), bearing a D70Y and an E67G mutation, respectively, formed prominent replication compartments that showed no association with TRIM69 filaments or accumulations (Fig. 9). Strikingly, the multimerization-defective mutants of TRIM69 (V95A L96A and L99A mutations), which did not exhibit antiviral activity and were ordinarily diffusely distributed in the cytosol (Fig. 4D), were nearly completely relocalized to replication compartments in VSV_IND_(NeonGreen/P)- or VSV_IND_(eGFP/P)-infected cells (Fig. 10A). Thus, TRIM69 recruitment to replication compartments was not itself sufficient to inhibit VSV_IND_ replication. Moreover, the dramatic redistribution of TRIM69(L99A) to replication compartments in VSV_IND_-infected cells was completely absent in VSV_IND_(eGFP/P)_TR1(D70Y)- and VSV_IND_(eGFP/P)_TR3(E67G)-infected cells (Fig. 10A). Together, these data indicate that specific sequences in P are necessary for the recruitment of TRIM69 to the VSV_IND_ replication machinery and vice versa.

To determine whether P was sufficient for TRIM69 recruitment, we expressed eGFP/P by transfection, in the absence of any other viral proteins. In contrast to the situation in VSV_IND_-infected cells, eGFP/P expressed alone was diffusely distributed throughout the cytoplasm (Fig. 10B). However, in TagRFP/TRIM69-expressing cells, eGFP/P was recruited to the TagRFP/TRIM69 accumulations and the two proteins colocalized extensively (Fig. 10B and C). Again, this colocalization was dependent on the viral determinant of TRIM69 sensitivity, as there was no colocalization between TagRFP/TRIM69 and eGFP/P(E67G) (Fig. 10B and C).

To determine whether P physically associated with TRIM69, we generated glutathione *S*-transferase (GST) proteins fused to a region of P encompassing the LSR domain (amino acids 32 to 107). We also constructed GST/P fusion protein-containing mutant LSR domains from VSV_IND_(eGFP)_TR1(E69K) and from VSV_IND_(eGFP/P)_TR3(E67G) (Fig. 8A). The GST/P fusion proteins were coexpressed with Cherry/TRIM69 in 293T cells and then purified from cell lysates with glutathione-Sepharose. Cherry/TRIM69 was nearly undetectable in clarified cell lysates, presumably due to its propensity to form higher-order multimers that were poorly soluble in nondenaturing detergents (Fig. 10D). Nevertheless, GST/P coprecipitated Cherry/TRIM69 such that it was highly enriched in precipitated fractions (Fig. 10D). Conversely, GST/P proteins bearing the TR mutations (E69K or E67G) did not precipitate detectable amounts of Cherry/TRIM69 (Fig. 10D). Thus, the isolated LSR domain was sufficient for the physical association of P with TRIM69.

### Mechanism of VSV replication inhibition by TRIM69.

The aforementioned data provide evidence that TRIM69 physically associates with P and ultimately inhibits VSV_IND_ replication compartment formation. However, the assembly of replication compartments requires multiple prior steps, each of which is a potential target of TRIM69. For example, because TRIM proteins can act as ubiquitin ligases (12), it was conceivable that TRIM69 might mediate the destruction of one or more viral proteins. Alternatively, interaction with P could block replication through a nondegradative mechanism by inhibiting (i) an initial pioneer round of transcription that employs the incoming virion RNA genome as a template to generate viral mRNAs, (ii) the translation of these new viral mRNAs to generate N, P, and L proteins, or (iii) the assembly of newly synthesized N, P, and L proteins with full-length negative-strand RNAs.

**FIG 11:**
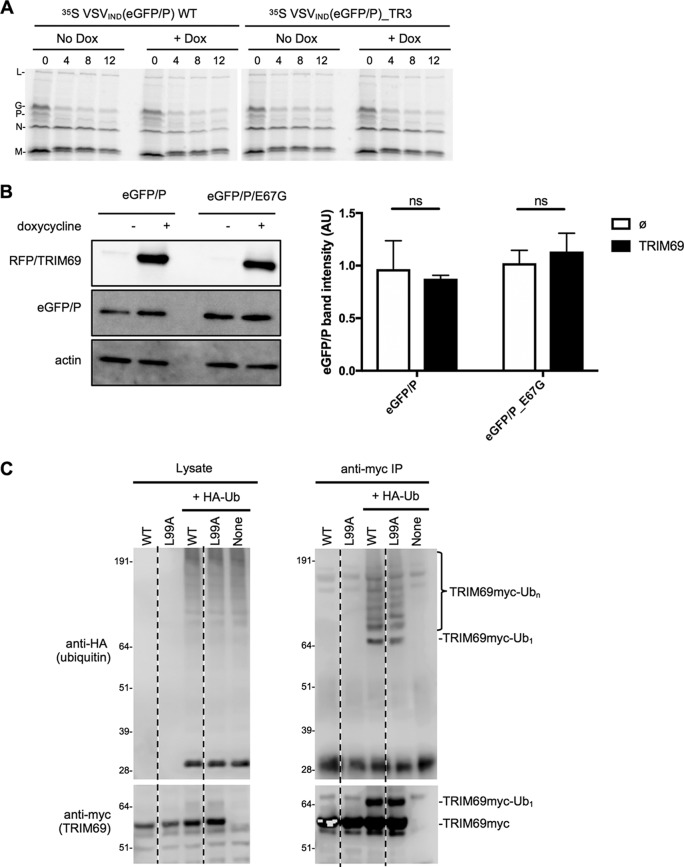
Lack of evidence for TRIM69-induced VSV_IND_ protein degradation as a mechanism of antiviral activity. (A) HT1080-TagRFP/TRIM69 cells were seeded and simultaneously treated or not with doxycycline. Sixteen hours later, cells were treated with cycloheximide and infected with ^35^S-radiolabeled VSV_IND_(eGFP) or VSV_IND_(eGFP/P)_TR3(E67K) at an MOI of 10. Cells were harvested at 0, 4, 8, and 12 h.p.i., and proteins were analyzed by SDS-PAGE. (B) Western blot analysis of eGFP/P protein levels in transfected HT1080-TagRFP/TRIM69 cells that were treated or not with doxycycline. Left, representative blot results; right, quantification of eGFP/P band intensities (mean values ± SD, *n* = 4). (C) TRIM69 autoubiquitination in 293T cells cotransfected with plasmids expressing TRIM69/myc and HA-ubiquitin. Lysates (left) and anti-myc antibody immunoprecipitates (right) were probed with anti-myc and anti-HA tag antibodies.

To determine whether TRIM69 targeted incoming viral proteins for degradation, we first infected cells with ^35^S-labeled virions at a high MOI and monitored the levels of virion proteins in the presence of cycloheximide (CHX) to prevent new protein synthesis and VSV replication. No substantial difference in the decay of incoming viral proteins in the presence or absence of TRIM69 was detected (Fig. 11A). Next, we monitored the levels of P protein following transfection into cells in which TRIM69 expression was or was not induced. Despite obvious recruitment of eGFP/P to sites of TRIM69 concentration (Fig. 10B), the overall levels of WT and TR mutant eGFP/P were equivalent and unaffected by TRIM69 (Fig. 11B). Finally, we found that WT and inactive (L99A) mutants of TRIM69 exhibited approximately equivalent levels of autoubiquitination (Fig. 11C). Overall, we found no evidence that TRIM69 drives VSV protein degradation, and the ubiquitin ligase activity of TRIM69 was unable to account for its antiviral activity.

**FIG 12:**
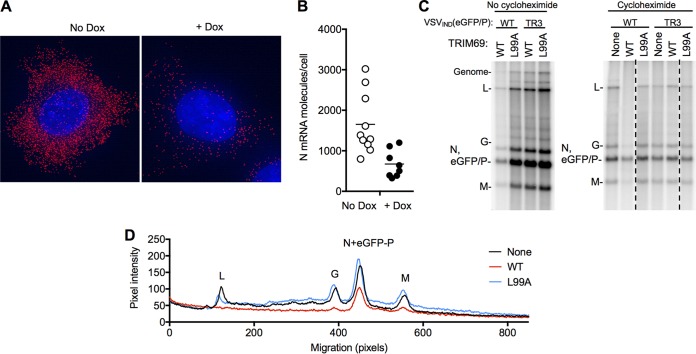
TRIM69 inhibits primary VSV_IND_ transcription. (A) Single-molecule FISH analysis of HT1080-TRIM69/myc cells treated with cycloheximide and infected with VSV_IND_(GFP/P) for 3 h, using probes directed to N mRNA. Representative individual cells are shown. (B) Quantification of single-molecule FISH analysis of HT1080-TRIM69 cells as described in the legend to panel A. Each symbol represents the result for an individual cell, plotted according to the number of N mRNA molecules in the cell. Unpaired *t* test, *P* = 0.0036. (C) HT1080 cells expressing WT or mutant (L99A) TagRFP/TRIM69 and cultured with actinomycin D and [^32^P]orthophosphate were infected with VSV_IND_(eGFP/P) or VSV_IND_(eGFP/P) (E67G) at an MOI of 100. To prevent genome replication, cells were also treated with cycloheximide as indicated. RNA was extracted at 5 h.p.i. and analyzed on an agarose-urea gel. Note that N and eGFP/P mRNAs comigrate. (D) Quantification of pixel intensities versus migration for the leftmost three lanes of the right panel in panel C, extracted using ImageJ.

Next, we quantified new viral mRNA (pioneer) transcription from incoming virion RNA templates in cells treated with CHX to prevent protein synthesis and replication. A single-molecule FISH assay and a pool of oligonucleotide probes directed at the plus-strand N mRNA (Table 2) demonstrated a clear reduction in pioneer N mRNA transcription in cells expressing TRIM69 (Fig. 12A and B). In an alternative approach to measure VSV transcription, we labeled target cells in the presence of actinomycin D (to block host mRNA synthesis) with [^32^P]orthophosphate. At 5 h after VSV_IND_(eGFP/P) infection, transcripts corresponding to L, G, and M mRNAs were clearly detectable (Fig. 12C). Transcripts corresponding to N and/or eGFP/P mRNAs were also detected but could not be distinguished from each other due to comigration (Fig. 12C). As expected, in the absence of CHX, mRNA synthesis and genome replication of VSV_IND_(eGFP/P) were inhibited by TRIM69 but not TRIM69(L99A), while VSV_IND_(eGFP/P)_TR3(E67K) mRNA synthesis and genome replication were insensitive to TRIM69. Importantly, when CHX was used to prevent protein synthesis and RNA replication, the presence of TRIM69 reduced the levels of all nascent VSV_IND_ mRNA transcripts (Fig. 12C). The magnitude of the effect on pioneer transcript levels appeared greatest for the L mRNA, least for the N and P mRNAs, and of intermediate magnitude for M and G mRNAs (12C and D). The multimerization-defective TRIM69(L99A) mutant did not show an effect on pioneer mRNA synthesis (Fig. 12C). Thus, TRIM69 inhibited primary transcription of the incoming VSV_IND_ virion RNA, with apparently greater effect on genes encoded near the 5′ end of the negative-strand genome.

## DISCUSSION

A number of ISGs, including Mx1, PKR, IFITM3, and tetherin, have previously been reported to inhibit VSV replication (22–25). However, it was not known whether this list represents a complete catalogue of ISG proteins with anti-VSV activity. We found that a number of antiviral ISGs contribute to the induced antiviral state that prevents VSV_IND_ replication in IFN-α-treated cells. Among these, we found that TRIM69 has a previously undescribed mechanism of action, inhibiting VSV_IND_ transcription by targeting the polymerase cofactor, P.

TRIM69 joins a growing list of TRIM proteins that have been shown to exhibit antiviral activity through various mechanisms (12). During the course of this work, TRIM69 itself was reported to inhibit dengue virus type 2 (DENV2) replication, albeit via a different mechanism from that described herein, namely, ubiquitin-induced degradation of the viral NS3 RNA helicase (26). While the manuscript was in preparation, another group also found that TRIM69 inhibits VSV_IND_ replication (27) but were unable to reproduce the reported finding of TRIM69 antiviral activity against DENV2 (26). These authors also demonstrated that P is a crucial determinant of TRIM69 sensitivity and showed that *TRIM69* exhibits signatures of positive selection, a frequent characteristic of antiviral genes (27). Other examples of antiviral TRIM proteins include TRIM5α, which inhibits early stages of retroviral infection by binding in a polyvalent manner to incoming retroviral capsids, promoting premature uncoating and degradation of virion components (28, 29). Another TRIM protein, TRIM25, has been reported to promote ubiquitination of the RNA sensor RIG-I, thereby inducing binding to mitochondrial antiviral signaling (MAVS) protein and stimulation of IFN production (30), although this model has recently been challenged (31). TRIM25 is also an important cofactor of the zinc finger antiviral protein (ZAP), which senses and depletes CG-rich viral RNAs, although the mechanism that enables ZAP activity remains unclear (32). A variety of other TRIM proteins have been reported to inhibit viral replication directly or indirectly through less well characterized mechanisms (12).

In the two aforementioned examples, high-order multimerization is crucial for activity. TRIM5 higher-order multimerization, driven by a B-box domain, facilitates the formation of a hexagonal lattice on the surface of incoming retroviral capsid, enabling polyvalent interaction between the capsid hexagonal lattice and a complementary hexagonal TRIM5 lattice (16, 33). In this case, higher-order multimerization results in a more avid interaction between TRIM5 and its viral capsid protein target. For TRIM25, RING domain dimerization enables engagement of ubiquitin-conjugated E2 enzymes and higher-order assembly of the RIG-I signalosome (17). Herein, we found that the RING domain dimer interface, analogous to that found in TRIM25, was required for higher-order TRIM69 multimerization, the formation of TRIM69 filaments, and antiviral function. However, abolition of high-order TRIM69 multimerization by mutation of the RING domain dimer interface did not prevent recruitment into VSV_IND_ replication compartments. Rather, recruitment of dimeric TRIM69 to replication compartments remained efficient but was inconsequential to VSV replication. Thus, for TRIM69, RING domain-mediated multimerization appeared to be required for antiviral activity but not target recognition. As RING domain dimerization might lead to E2 recruitment, as well as high-order multimer formation, it is not clear whether higher-order multimerization *per se* or downstream E2 recruitment is essential for TRIM69 activity. However, the lack of effect of TRIM69 on incoming virion protein stability or on coexpressed P levels, coupled with the finding that the L99A mutant maintained ubiquitin ligase activity, argues that destruction of virion proteins is not central to the mechanism of action of TRIM69. Unfortunately, we were not able to identify a TRIM69 mutant that maintained higher-order multimer formation but abolished ubiquitination activity.

We did not formally demonstrate that TRIM69 directly binds to P, and it is possible that P interacts with some bridging host protein(s) that is(are) bound by TRIM69. However, a direct interaction between TRIM69 and the LSR domain of P is the most likely molecular event underlying recognition and disruption of the viral transcription/replication machinery. P is required for the interaction between L and the N-coated RNA template (20, 34) and, thus, for initial transcription following viral entry, as well as for the formation of replication compartments (10). Given that P plays a pivotal, multifactorial role in VSV RNA synthesis and has no cellular homologs, it represents an attractive target for intrinsic immune defenses. Moreover, the pioneer round of transcription may represent a point of vulnerability when the number of viral targets is low and ISG-mediated inhibition might exert maximal effects. Nevertheless, subsequent rounds of transcription are thought to proceed by essentially the same mechanism as pioneer transcription, and it is possible that TRIM69 inhibits transcription at all stages of VSV_IND_ RNA accumulation within an infected cell. With the caveat that overexpressed TRIM69 was used, we noted the formation of elongated filamentous accumulations of P, coincident with TRIM69 filaments in TRIM69-expressing cells, rather than the spherical droplets that normally characterize the phase-separated VSV replication compartments. This suggests that TRIM69 might inhibit replication compartment formation, in addition to its effects on transcription. Because the L99A TRIM69 mutant retained the ability to be recruited by P and localizes to the phase-separated replication compartments and yet did not inhibit VSV_IND_ transcription, it appears that the interaction of TRIM69 with the LSR of P does not prevent functional P-L complex formation. While further study will be required to elucidate the molecular details of how TRIM69 recognizes and disrupts the VSV replication machinery, these findings reveal a new facet of the diverse ways in which IFNs control the replication of viruses.

## MATERIALS AND METHODS

### Cells.

HT1080 cells were obtained from ATCC and maintained in Dulbecco’s modified Eagle’s medium (DMEM) (catalog number 11995065; Thermo Fisher) with 10% fetal calf serum (FCS) (catalog number F8067-500ML; Sigma) and gentamicin (catalog number 15710064; Thermo Fisher). An IFN-α (catalog number 11200-2; PBL Assay Science)-sensitive HT1080 single-cell clone was derived by limiting dilution. BHK21 cells obtained from ATCC were maintained in Eagle’s minimum essential medium (EMEM) (catalog number 30-2003; ATCC) supplemented with 10% FCS (catalog number F8067-500ML; Sigma) and gentamicin (catalog number 15710064; Thermo Fisher). Cells were not tested for mycoplasma contamination but were periodically tested for retrovirus contamination using a PCR-based reverse transcriptase assay (35, 36).

BSRT7 cells (a kind gift from K. Conzelmann) (37) and African green monkey kidney Vero cells (CCL-81; ATCC) were maintained in DMEM (catalog number 10-013-CV; Corning, Inc.) containing 10% fetal bovine serum (FBS) (catalog number TCB 101; Tissue Culture Biologicals) at 37°C and 5% CO_2_.

### Plasmid construction.

The pLKO-derived doxycycline-inducible lentiviral expression vector was used as previously described (3). pLKO TagRFP or mScarlet N-terminally tagged human TRIM69 constructs comprising various species and mutants were cloned by overlapping PCR using SfiI restriction sides. pLKO myc-TRIM69 or mutant constructs were cloned using a forward primer containing a myc tag and SfiI restriction sites. Plasmids expressing WT or mutant TRIM69-3×myc were generated in the pCR3.1 expression vector containing an EcoRI-XhoI-NotI multiple cloning site, followed by the addition of an in-frame 3×myc tag using EcoRI and XhoI restriction sites. Plasmids expressing WT or mutant Cherry-TRIM69 were generated in the pCR3.1 Cherry EcoRI-XhoI-NotI background using EcoRI and XhoI restriction sites.

pCAGGS-eGFP/P_TR3(E67G) was derived from pCAGGS-eGFP/P (38) and was made by site-directed mutagenesis using Q5 high-fidelity DNA polymerase (catalog number M0491; New England Biolabs) and primers ATTGAAGACAATCAAGGCTTGTATG and TTGATTGTCTTCAATACCTGGTTCAGATTCTGTGTCAGAAT.

Sequences encoding VSV-P WT and E67G and E69K mutants (amino acids 32 to 107) were amplified from pCAGGS-eGFP/P plasmids and inserted in frame with the glutathione-*S*-transferase (GST) gene in the pCAGGS-GST expression plasmid using the following restriction enzymes and oligonucleotide primers: 5′ EcoRI and GAGGAGGAATTCGCTGAAAAGTCCAATTATGAGTTG and 3′ XhoI and CTCCTCCTCGAGCTAGTCCGAAGTAAATACAACATCCAC.

The plasmids used to produce Sendai virus were kindly provided by Benhur Lee. The recombinant Sendai virus (rSeV) clone provided contained eGFP and mutations in the F and M genes to allow trypsin-independent growth. A hammerhead ribozyme (Hh-Rbz) sequence was present between the T7 promoter and the start of the viral antigenome to enhance the rescue efficiency (39). The GFP gene that had been positioned between the N and P genes via duplication of the N-P intergenic region was replaced with nLuc. Briefly, the N and P regions were amplified with forward primer Pre-SbfI-for (5′-TGACCATGATTACGCCAAGCTTAA-3′) and reverse primer nLuc_SeV_rev (5′-GAAATCTTCGAGTGTGAAGACCATGCGGTAAGTGTAGCCGAAGCCGTG-3′) and forward primer nLuc-SeV_for (5′-CTGTGCGAACGCATTCTGGCGTAATGAGATAGGAGGAATCTAGGATCA-3′) and reverse primer Post-SmaI-6860-rev (5′-GATGGTAGATTGGGTCTCTCTGTG-3′), respectively. The nLuc gene (Promega) was amplified with forward primer SeV-nLuc_for (5′-CACGGCTTCGGCTACACTTACCGCATGGTCTTCACACTCGAAGATTTC-3′) and reverse primer SeV-nLuc_rev (5′-TGATCCTAGATTCCTCCTATCTCATTACGCCAGAATGCGTTCGCACAG-3′). After overlap extension PCR using the outermost primers, the fragments were inserted into the Sbf1- and SmaI-digested rSeV_GFP construct using Gibson assembly.

pVSV_NJ_(+)-eGFP was constructed as described previously for plasmid pVSV1(+), encoding VSV_IND_ genomic RNA (40). Briefly, pVSV_NJ_(+)-eGFP was assembled from plasmid made by reverse transcription-PCR of each of the VSV_NJ_ genes of the Ogden strain and intergenic junctions by standard cloning techniques. These clones were assembled into a full-length cDNA and inserted between the bacteriophage T7 promoter and a cDNA copy of the self-cleaving ribozyme from the antigenomic strand of hepatitis D virus (HDV). The eGFP gene was inserted in the first position (in an additional transcription unit before N) as described for VSV_IND_ (41).

### Viruses.

Plasmids encoding the full-length VSV_IND_ genome (pVSV-FL), as well as individual VSV_IND_ N, P, L, and G genes, were purchased from Kerafast [VSV-FL+(2) VSV plasmid expression vector system, catalog number EH1002] or were generated as described previously (40). VSV_IND_ viruses were generated by infecting 293T cells with T7-expressing vaccinia virus (vTF7-3) at an MOI of 5, followed by transfection with pVSV plasmids and plasmids encoding VSV N, P, L, and G under the control of a T7 promoter. Supernatants were harvested 48 h posttransfection (h.p.t.), filtered (0.2 μm) to remove the bulk of the vaccinia virus, and plaque purified on 293T cells. Plaque-purified virus was expanded on 293T cells, and cell culture supernatant was harvested, passed through a 0.2-μm filter, and frozen in aliquots. Virus titers (PFU/ml) were determined by plaque formation using HT1080 or BHK21 cells. VSV encoding nanoluciferase (nLuc) was generated by inserting the nLuc-encoding sequences (from pNL1.1; Promega) into the pVSV plasmid between the envelope and L genes, along with appropriate VSV regulatory sequences. VSV derivatives encoding mNeonGreen were generated by fusing the mNeonGreen-encoding sequence to the N terminus of P.

VSV_IND_(eGFP) (41), VSV_IND_(eGFP/P) (38), and RabV(eGFP-ΔG) (42) were described previously. VSV_NJ_(eGFP) was rescued from pVSV_NJ_(+)_eGFP by following a previously described protocol (40). Rhabdovirus stocks were grown on BSR-T7 cells, BHK cells, or Vero cells, and the titers determined by plaque assay on BSR-T7 or HT1080 cells. Briefly, cells were seeded in DMEM–10% FBS and infected 1 day later for 1 h with viruses at a multiplicity of infection (MOI) of 0.01. Virus suspensions were replaced with DMEM–2% FBS, and cell supernatants were harvested when 95% of the cells were infected and ready to detach (about 24 h for VSV_IND_). For RabV(eGFP-ΔG)-G_VSVind_, cells were transfected 8 h prior to infection with plasmid expressing VSV_IND_-G using Lipofectamine 2000 (catalog number 11668-019; Invitrogen). For concentrated sucrose cushion-purified virus stocks, infected cell supernatant was concentrated through a 15% sucrose cushion in NTE (10 mM Tris-HCl, pH 7.4, 100 mM NaCl, 1 mM EDTA) at 110,000 × *g* for 2 h at 4°C. Pellets were resuspended overnight at 4°C in NTE.

Rescue of replication-competent Sendai virus from transfected plasmids was done as previously described (39) with transfection into 293T cells using Lipofectamine LTX (catalog number 15338100; Invitrogen) according to the manufacturer’s recommendations. Virus titers (PFU/ml) were determined by plaque formation using HT1080 target cells.

### siRNA-based ISG screen.

The 400 most-IFN-α-inducible ISGs were chosen using a compilation of microarray data from 293T, HT1080, CEM, Jurkat, MT2, MT4, C8166, Hut R5, H9, Sup T1, U937, THP-1, K562, HL60, and KG1a cells (Table 1). All ISG screens were conducted in a 96-well format. Amounts of 3 × 10^3^ HT1080 cells were plated, transfected with siRNA SMARTpools (Dharmacon) using RNAimax (catalog number 13778150; Invitrogen) on the following day, and treated with 10 U/ml of IFN-α (catalog number 11200-2; PBL Assay Science) at 8 h.p.t. The cells were infected with 30 PFU of VSV nLuc per well the next day. At 20 h postinfection (h.p.i.), cells were washed 3 times in 1× phosphate-buffered saline (PBS) and lysed in passive lysis buffer (catalog number E1941; Promega), and luciferase was measured using the Nano-Glo luciferase assay system (catalog number N1130; Promega) and a Modulus II multimode microplate reader (Turner BioSystems).

### siRNA experiments.

Amounts of 3 × 10^3^ HT1080 cells were plated in a 96-well plate, transfected with siRNA SMARTpools or the most efficient individual siRNA (Dharmacon) (Table 1), treated with increasing concentrations of IFN-α or a fixed dose of 10 U/ml of IFN-α (catalog number 11200-2; PBL Assay Science) at 8 h.p.t., and harvested as described above.

### Inducible expression of TRIM69.

Inducible HT1080 cells were generated by transduction with an pLKO-derived vector as described previously (3), followed by selection with 1.25 μg/ml puromycin (catalog number P8833-100MG; Sigma-Aldrich). Expression was induced in pLKO-transduced cell lines through an overnight treatment with 0.5 μg/ml doxycycline hyclate (catalog number 324385; Sigma-Aldrich) prior to viral challenge.

### Deconvolution and structured illumination microscopy (SIM).

Amounts of 3 × 10^4^ HT1080 cells were plated onto gelatin-coated, 8-chambered, no. 1.5 borosilicate glass-bottom slides (catalog number 155409; LabTek), and TagRFP/TRIM69 or mScarlet/TRIM69 expression was induced by overnight treatment with 0.5 μg/ml doxycycline hyclate (Sigma-Aldrich). Cells were infected with mNeonGreen/P or eGFP/P VSV at an MOI of 3, fixed 4 h.p.i. using 4% formaldehyde (catalog number P6148-1KG; Sigma), and imaged by deconvolution microscopy (DeltaVision OMX SR imaging system). All images were generated by maximum-intensity projection using the Z project function in ImageJ (version 2.0.0-rc-59/1.51w).

### Confocal microscopy.

HT1080 TagRFP/TRIM69 cells were seeded onto a 1.5-mm coverslip (catalog number CS-12R15; Warner Instruments) in a 24-well plate and simultaneously treated or not with 0.5 μg/ml doxycycline. Sixteen hours later, cells were transfected or not with pCAGGS-eGFP/P and pCAGGS-eGFP/P(E67G) plasmids with Lipofectamine 2000 (catalog number 11668-019; Invitrogen), following the manufacturer’s protocol. Twenty-four hours posttransfection, cells were washed with 500 μl Dulbecco’s PBS (DPBS) (catalog number 59300C; Sigma), fixed for 15 min at room temperature with 250 μl DPBS containing 2% paraformaldehyde, washed twice with 1 ml DPBS containing 10 mM glycine (to quench residual paraformaldehyde), stained for 15 min with DAPI (4′,6-diamidino-2-phenylindole) diluted in DPBS containing 0.5% bovine serum albumin, and washed twice with 1 ml DPBS containing 0.05% Tween 20 and once with 1 ml H_2_O. Coverslips were mounted onto slides with 4 μl ProlongGold (catalog number P36930; Invitrogen). Confocal images were acquired on a Nikon T1 inverted microscope equipped with a Yokogawa CSU-W1 scan head, a Toptica laser launch, and an Andor Zyla 4.2 plus sCMOS (scientific complementary metal-oxide semiconductor) camera using a plan apochromat lambda 100×/1.45 numeric aperture (NA) differential inference contrast (DIC) oil objective. The acquisition software was NIS Elements AR 5.02. The emitted light from eGFP and TagRFP fluorophores was collected using a Semrock multi-band-pass dichroic filter (Di01-t 405/488/561/647) and Chroma 525/36 and 605/52 band-pass emitters, respectively.

### smFISH.

Single-molecule fluorescent *in situ* hybridization (smFISH) probes against both the plus and minus strands of VSV N were designed using the Stellaris Probe Designer, version 2.0 (Biosearch Technologies) (Table 2). For each RNA strand, 41 (plus strand) or 42 (minus strand) oligonucleotide probes were synthesized by IDT to contain a 5′ amino modifier (C6). The 5′ amino-modified probes for each RNA were resuspended to 1.25 μg/ml, pooled, and purified by three chloroform extractions followed by ethanol precipitation. Then, 50-μg amounts of the pooled probes were labeled with ester-modified Alexa Fluor 488 or Alexa Fluor 549 using the Alexa Fluor 488 oligonucleotide amine labeling kit (catalog number A20191; Thermo Fisher). After labeling, the pooled probes were ethanol precipitated, resuspended in RNase-free water, and purified via the Oligo Clean & Concentrator kit from Zymo Research (catalog number D4060). The pooled probes were eluted in RNase-free Tris-EDTA (TE), pH 8.0 (catalog number AM9849; Ambion), and adjusted to a final concentration of 12.5 μM. For FISH, 3 × 10^4^ HT0180-myc/TRIM69 cells were seeded onto gelatin-coated, 8-chambered, no. 1.5 borosilicate glass-bottom slides (catalog number 155409; LabTek). Doxycycline-treated or untreated cells were pretreated for 30 min with 100 μg/ml of cycloheximide (catalog number C4859; Sigma-Aldrich) and infected at an approximate MOI of 20 with VSV(mNeonGreen/P) virus. At 2 h 45 min postinfection, the cells were washed with PBS (catalog number AM9624; Ambion) and fixed with 4% formaldehyde (catalog number 28908; Thermo Fisher) in PBS for 30 min at room temperature (RT). Following permeabilization with 70% ethanol for 2 h at RT, the cells were washed with Stellaris RNA FISH wash buffer A (catalog number SMF-WA1-60; Biosearch Technologies) for 5 min at RT. The cells were probed for N or P plus- or minus-strand RNA with 0.125 μM Alexa Fluor 488- or Alexa Fluor 549-labeled probes in Stellaris RNA FISH hybridization buffer (catalog number SMF-HB1-10; Biosearch Technologies) for 16 to 18 h at 37°C. The cells were then washed two times for 30 min at 37°C in Stellaris RNA FISH wash buffer A (catalog number SMF-WA1-60; Biosearch Technologies); the second wash contained Hoechst stain at 1 μg/ml. After a 5-min wash with Stellaris RNA FISH wash buffer B (catalog number SMF-WB1-20; Biosearch Technologies), the cells were rinsed three times with PBS and imaged by deconvolution microscopy (DeltaVision OMX SR imaging system). All images were generated by maximum intensity projection using the Z project function in ImageJ (version 2.0.0-rc-59/1.51w). RNA spots were quantified using StarSearch, developed by the Raj laboratory (https://www.seas.upenn.edu/~rajlab/StarSearch/launch.html).

### VSV replication assays.

Amounts of 1 × 10^4^ HT1080 cells were plated in a 96-well plate format, and TRIM69, Mx1, CD68, or RFP expression was induced by overnight treatment with 0.5 μg/ml doxycycline hyclate (Sigma-Aldrich). The cells were infected with 30 PFU of VSV(nLuc) per well the next day. At 20 h.p.i. or time points indicated in Fig. 2, supernatant was collected, cells were lysed in passive lysis buffer (catalog number E1941; Promega), and luciferase was measured using the Nano-Glo luciferase assay system (catalog number N1130; Promega) and Modulus II multimode microplate reader (Turner BioSystems), or the viral titers in supernatant containing virions were determined on BHK21 cells under a methyl cellulose overlay.

To compare the growth of the TR viruses, 1.2 × 10^6^ Vero cells were infected with the different viruses at an MOI of 0.05. Aliquots of the supernatants were harvested at 8, 12, 16, 20, and 24 h.p.i., and the titers determined by cytometry on BSR-T7 cells. Titers are expressed in number of infectious units per ml, i.e., the number of virions leading to detectable expression of eGFP in BSR-T7 cells per ml.

For short-term (single-cycle) infection assays, HT1080-TagRFP/TRIM69 cells were seeded and simultaneously treated or not with 0.5 μg/ml doxycycline. Sixteen hours later, cells were infected with VSV_IND_(eGFP), VSV_NJ_(eGFP), or RabV(eGFP-ΔG)-G_VSVind_ at an MOI of 1 for 1 h. Six hours after infection, TagRFP/TRIM69 and eGFP expression levels were monitored by epifluorescence microscopy.

### Selection of TRIM69-resistant (TR) viruses.

TRIM69-resistant VSV_IND_(eGFP) and VSV_IND_(eGFP/P) were selected by plaque assay on HT1080-TagRFP/TRIM69 cells. Cells were seeded and simultaneously treated with 0.5 μg/μl doxycycline (catalog number D9891; Sigma). Sixteen hours later, cells were infected for 1 h with 1:10 dilutions of the viral stocks and overlaid with medium containing 0.25% agarose. Plaques were picked and amplified once on HT1080 cells expressing TagRFP/TRIM69 and then on BSR-T7 cells.

### Western blotting.

For the experiments whose results are shown in Fig. 2A, 3B, 4E, 10D, and 11C, cells were lysed in lithium dodecyl sulfate (LDS) sample buffer (catalog number NP0008; Invitrogen) and proteins were separated by electrophoresis on NuPAGE 4 to 12% bis-Tris gels (catalog number NP0323BOX; Invitrogen) and blotted onto nitrocellulose membranes (catalog number 10600003; GE Healthcare). Membranes were incubated with rabbit anti-heat shock protein 90 (HSP90) antibody (catalog number 13171-1-AP; Proteintech), mouse anti-VSV-M antibody (catalog number EB0011; Kerafast), mouse anti-RFP antibody (catalog number ab125244; Abcam), mouse anti-myc antibody (catalog number 904401; Biolegend), rabbit anti-TRIM69 antibody (catalog number 12951-1-AP; Proteintech), or rabbit anti-CD68 antibody (catalog number 25747-1-AP; Proteintech). Thereafter, membranes were incubated with goat anti-rabbit antibody–IRDye 800CW and goat anti-mouse antibody–IRDye 680RD (catalog numbers 926-32211 and 926-32220, respectively; LI-COR Biosciences) and scanned using a LI-COR Odyssey infrared imaging system. Alternatively, membranes were incubated with appropriate horseradish peroxidase (HRP)-conjugated secondary antibodies (goat anti-mouse and goat anti-rabbit antibodies, catalog number 15-035-174 and catalog number 111-035-144, respectively; Jackson ImmunoResearch) and visualized using SuperSignal West Femto chemiluminescence solution (catalog number PI34095; Thermo Fisher) and a C-DiGit Western blot scanner (LiCor).

For the experiment whose results are shown in Fig. 11B, cells were lysed in 50 μl of 20 mM Tris-HCl, pH 8, 150 mM NaCl, 0.6% NP-40, 2 mM EDTA, and 1× cOmplete protease inhibitor cocktail (catalog number 4693116001; Roche). Soluble proteins were separated on 10% acrylamide gels, transferred onto nitrocellulose membranes, and incubated with mouse anti-RFP antibody (catalog number ab125244; Abcam), rabbit anti-GFP antibody (catalog number ab6556; Abcam), or mouse antiactin antibody (catalog number A5316; Sigma), followed by incubation with HRP-conjugated anti-mouse antibody (catalog number 31430; Invitrogen) or anti-rabbit antibody (catalog number A0545; Sigma). HRP activity was visualized using the Pierce ECL Western blotting kit (catalog number 32209; Thermo Scientific) and imaged with an Amersham Imager 600 (GE Healthcare). Protein band intensities were quantified using ImageJ software.

### GST coprecipitation assay.

Approximately 4 million 293T cells were transfected via polyethylenimine (catalog number 23966; Polysciences) with 5 μg of glutathione *S*-transferase (GST) or GST/P(32–107) (WT or E69K or E67G mutant) and 5 μg Cherry or Cherry/TRIM69 (WT or L99A). Two days posttransfection, the cells were lysed on ice for 10 min in 1 ml 50 mM Tris, pH 7.4, 150 mM NaCl, 1% digitonin. Lysates were cleared by centrifugation and incubated with 25 μl glutathione-Sepharose 4B beads (catalog number 17075601; GE Healthcare) for 3 h at 4°C. The beads were washed 3 times in 1 ml lysis buffer and eluted by boiling in 50 μl 1× SDS sample buffer. The eluted proteins were separated on NuPAGE 4 to 12% bis-Tris protein gels (catalog number NP0323BOX; Thermo Fisher) and transferred onto nitrocellulose (catalog number 10600003; GE Healthcare) for probing with rabbit anti-GST antibody (catalog number 19256; Abcam) and mouse anti-RFP antibody (catalog number 125244; Abcam), followed by goat anti-rabbit antibody–IRDye 680 (catalog number 926-68071; LiCor) and goat anti-mouse antibody–IRDye 800 (catalog number 926-32210; LiCor).

### Measurement of TRIM69 multimerization.

Inducible HT1080 cells (3 × 10^6^) expressing wild-type or mutant TagRFP/TRIM69 were collected in 1× PBS and cross-linked by treatment with 0.2 mM EGS [ethylene glycol bis(succininic acid *N*-hydroxysuccinimide ester)] (catalog number 13308-100; CovaChem), a membrane-permeable cross-linker. After 30 min of incubation at room temperature, cells were lysed in LDS sample buffer (Invitrogen) and proteins were separated by electrophoresis on NuPAGE 4 to 12% bis-Tris gels (catalog number NP0323BOX; Invitrogen) and blotted onto nitrocellulose membranes (catalog number 10600003; GE Healthcare). Blots were probed with rabbit anti-HSP90 antibody (Proteintech) and mouse anti-RFP antibody (catalog number 125244; Abcam). Thereafter, membranes were incubated with goat anti-rabbit antibody–IRDye 800CW and goat anti-mouse antibody–IRDye 680RD (catalog number 926-32211 and catalog number 926-32220, respectively; LI-COR Biosciences) and scanned using a LI-COR Odyssey infrared imaging system.

### TRIM69 autoubiquitination.

Amounts of 7 × 10^5^ 293T cells were transfected via polyethylenimine (catalog number 23966; Polysciences) with 1 μg of pCR3.1 TRIM69-3×myc and 500 ng of pHA-ubiquitin. At 36 h.p.t., cells were thoroughly lysed at room temperature in detergent-rich radioimmunoprecipitation assay (RIPA) buffer (50 mM Tris, pH 7.4, 150 mM NaCl, 1 mM EDTA, 1.0% glycerol, 0.5% SDS, supplemented with protease inhibitor [catalog number 04693159001; Roche] and 5 mM *N*-ethylmaleimide [catalog number 04259-5G; Sigma] to inhibit deubiquitination), sonicated, and cleared of cellular debris by microcentrifugation. Lysates were transferred into fresh Eppendorf tubes and diluted 5-fold in the same buffer containing NP-40 rather than SDS, to adjust the concentration of SDS to 0.1% and NP-40 to 1.0%. Lysates were incubated with 30 μl Dynabeads (catalog number 10001D; Invitrogen) for 2 h at 4°C. The beads were washed 3 times in 1 ml lysis buffer and eluted by boiling in 50 μl 1× SDS sample buffer. The eluted proteins were separated on NuPAGE 4 to 12% bis-Tris protein gels (catalog number NP0323BOX; Thermo Fisher), transferred onto nitrocellulose (catalog number 10600003; GE Healthcare) for probing with mouse anti-myc antibody (Biolegend catalog number 904401) and rabbit antihemagglutinin (anti-HA) antibody (catalog number 51064-2-AP; Proteintech), followed by goat anti-rabbit antibody–IRDye 800CW and goat anti-mouse antibody–IRDye 680RD (catalog number 926-32211 and catalog number 926-32220, respectively; LI-COR Biosciences), and scanned using a LI-COR Odyssey infrared imaging system.

### Radiolabeling and analysis of virion proteins.

For production of virions containing radiolabeled proteins, BSR-T7 cells were seeded in a 150-mm dish in DMEM–10% FBS. The next day, cells were incubated with 4 ml methionine-free, cysteine-free DMEM (catalog number 17-204-Cl; Corning, Inc.) for 30 min and infected for 1 h at an MOI of 3 in 4 ml methionine-free, cysteine-free DMEM. The virus solution was then replaced with 12 ml methionine-free, cysteine-free DMEM containing 120 μl of EXPRE35S35S protein labeling mixture (catalog number NEG072007MC; PerkinElmer). Twenty hours later, cell supernatants were harvested and sucrose cushion purified, and the virus titers determined by plaque assay.

To monitor virion protein decay, TagRFP/TRIM69 cells were seeded in a 12-well plate and simultaneously treated or not with 0.5 μg/ml doxycycline. Sixteen hours later, cells were treated with 100 μg/ml cycloheximide and infected for 1 h with ^35^S-radiolabeled viruses at an MOI of 10. Cells were washed twice with DMEM–2% FBS and incubated in 0.5 ml DMEM–2% FBS containing 100 μg/ml cycloheximide. At 0, 4, 8, and 12 h.p.i., cells were harvested, washed in DPBS, and lysed with 20 μl Rose lysis buffer (50 mM Tris-HCl, pH 7.4, 5 mM EDTA, 150 mM NaCl, 1% NP-40, 1× cOmplete protease inhibitor cocktail [catalog number 4693116001; Roche]). Proteins were analyzed by SDS-PAGE, gels were fixed in 30% methanol and 10% acetic acid, dried, and exposed overnight to a phosphor screen, and the radiolabeled proteins were visualized using a Typhoon FLA 9500 scanner. Protein band intensities were quantified with ImageJ software.

### Radiolabeling and analysis of primary transcripts.

HT1080-TagRFP/TRIM69 and HT1080-TagRFP/TRIM69(L99A) cells were seeded in 6-well plates and simultaneously treated or not with 0.5 μg/ml doxycycline. Sixteen hours later, cells were incubated in phosphate-free DMEM (catalog number 11971-025; Gibco) for 30 min, followed by a 30-min incubation in phosphate-free DMEM containing or not containing 0.5 μg/ml doxycycline, 10 μg/ml actinomycin D (catalog number A5156; Sigma), and 100 μg/ml cycloheximide (catalog number 94271; VWR). Cells were then infected for 30 min with sucrose cushion-purified virus at an MOI of 100. Virus solutions were replaced with 1 ml phosphate-free DMEM containing or not containing 0.5 μg/ml doxycycline, 10 μg/ml actinomycin D, and 100 μg/ml cycloheximide, and then 10 μl of phosphorus-32 radionuclide (catalog number NEX053H005MC; PerkinElmer) was added dropwise. Five hours postinfection, RNA was extracted using TRIzol reagent (catalog number 15596018; Invitrogen), following the manufacturer’s protocol. RNA was boiled at 100°C for 1 min, incubated on ice for 2 min, mixed with 1.33× loading buffer (33.3 mM citrate, pH 3, 8 M urea, 20% sucrose, 0.001% bromophenol blue), and analyzed on a 25 mM citrate, pH 3, 1.75% agarose, 6 M urea gel, running for 18 h at 4°C and 180 V. Gels were fixed in 30% methanol and 10% acetic acid, dried, and exposed overnight to a phosphor screen (GE Healthcare), and the radiolabeled RNA products were visualized using a Typhoon FLA 9500 scanner (GE Healthcare).
